# Genetic Evidence Suggesting Potential Associations between Sleep-Related Phenotypes and Psychiatric Disorders: A Two-Sample Mendelian Randomization Study

**DOI:** 10.1159/phg/aenag005

**Published:** 2026-09-30

**Authors:** Gahyun Kim, Minyoung Kim, Kyung-Hun Sung, Youngoh Bae, Seung Won Lee

**Affiliations:** a Department of Precision Medicine, Sungkyunkwan University School of Medicine, Suwon, Republic of Korea; b Department of MetaBioHealth, Sungkyunkwan University School of Medicine, Suwon, Republic of Korea; c Department of Internal Medicine, Seoul St. Mary’s Hospital, College of Medicine, The Catholic University of Korea, Seoul, Republic of Korea; d Department of Neurological Surgery, Asan Medical Center, University of Ulsan College of Medicine, Seoul, Republic of Korea; e Department of Family Medicine, Kangbuk Samsung Hospital, Sungkyunkwan University School of Medicine, Seoul, Republic of Korea

**Keywords:** Mendelian randomization, Sleep-related phenotypes, Psychiatric disorders

## Abstract

**Introduction:**

Sleep-related phenotypes, including insomnia symptoms, sleep apnea, and habitual sleep duration, are increasingly recognized as potentially modifiable factors associated with psychiatric disorders. However, their potential causal roles remain uncertain because observational studies are susceptible to confounding and reverse causation.

**Methods:**

Two-sample mendelian randomization (MR) was performed using self-reported insomnia symptoms, sleep apnea, and habitual sleep duration, as exposures against 10 psychiatric outcomes: ADHD, PTSD, MDD, PPD, BIP, ASD, OCS, SCZ, AN, and PD. GWAS summary statistics were obtained from the UK Biobank, Psychiatric Genomics Consortium, and affiliated consortia. Inverse-variance weighted (IVW) was the primary method, with MR-Egger, weighted median, and MR-PRESSO applied as sensitivity analyses. Multivariable MR (MVMR) was conducted to estimate independent associations of the sleep-related phenotypes modeled simultaneously.

**Results:**

Genetically predicted insomnia symptoms showed Bonferroni-significant associations with increased risk of ADHD (OR 2.50, 95% CI 1.65–3.78, *p* < 0.001) and PTSD (OR 1.32, 95% CI 1.12–1.56, *p* < 0.001). Nominal associations were observed for MDD (OR 1.57, 95% CI 1.16–2.12, *p* = 0.003), PPD (OR 2.35, 95% CI 1.07–5.13, *p* = 0.033), and BIP (OR 1.67, 95% CI 1.03–2.68, *p* = 0.036). Genetically predicted sleep apnea showed nominal associations with OCS (OR 1.20, 95% CI 1.07–1.35, *p* = 0.002) and AN (OR 1.57, 95% CI 1.17–2.11, *p* = 0.003). Genetically predicted longer sleep duration showed nominal associations with increased risk of BIP (OR 1.26) and SCZ (OR 1.67) but did not survive multiple-testing correction. MVMR analyses showed associations of longer sleep duration with ADHD (protective; OR 0.16, 95% CI 0.05–0.54, *p* = 0.004) and AN (OR 37.66, 95% CI 4.12–345.21, *p* = 0.001); however, these estimates were imprecise and potentially affected by conditional weak-instrument bias. Sensitivity analyses did not detect significant evidence of directional horizontal pleiotropy based on the MR-Egger intercept test in univariable analyses, although MVMR estimates remained imprecise.

**Conclusion:**

The sleep-related phenotypes showed heterogeneous patterns of association with psychiatric disorders. Genetically predicted insomnia symptoms were associated with the broadest range of psychiatric outcomes, whereas sleep apnea showed more outcome-specific associations. These findings highlight the importance of considering multiple dimensions of sleep when evaluating psychiatric disorders.

## Introduction

Mental illness is a major public health problem worldwide, not only reducing individuals’ quality of life but also causing a significant socioeconomic burden [1]. OECD (2025) pointed out that mental health problems pose a huge burden on the health, well-being, and economy of the population, especially with one in five adults suffering from depression and anxiety symptoms [2]. The economic impact of mental illness is significant, considering not only the direct medical costs but also indirect costs such as lost productivity and premature death. Anxiety disorders and depression alone are estimated to cause over USD 1 trillion in annual economic losses [3, 4]. Furthermore, mental illness often presents with a chronic course, placing a significant long-term burden on individuals, their families, and society as a whole. Given the marked heterogeneity of mental disorders in their clinical presentation and underlying pathophysiology, identifying upstream and potentially modifiable risk factors that contribute to psychiatric vulnerability has become a major public health priority [5].

Sleep-related phenotypes encompass both sleep disorders, such as insomnia and sleep apnea, and sleep traits, such as habitual sleep duration [6]. These phenotypes have been recognized as potentially modifiable factors associated with several psychiatric disorders [7–9]. Sleep is an essential physiological process for maintaining neurophysiological homeostasis, emotional regulation, and cognitive function, and abnormalities in sleep quality and quantity can directly affect brain function and mental health [10]. Accumulating evidence suggests that disturbances in these sleep dimensions may influence brain function and psychiatric vulnerability through distinct biological pathways [11, 12].

Observational studies have been conducted to elucidate the association between sleep characteristics and mental disorders; however, these findings are inherently limited by residual confounding and reverse causation, precluding robust causal inference [13]. Mendelian randomization (MR), which leverages genetic variants as instrumental variables (IVs), offers an opportunity to strengthen causal interpretation by mitigating these biases. Moreover, given the substantial correlation among different sleep-related phenotypes [14], multivariable MR (MVMR) enables the simultaneous modeling of multiple sleep phenotypes to estimate their independent effects [15].

Previous MR studies have reported associations between insomnia symptoms and several psychiatric disorders, including autism spectrum disorder (ASD), bipolar disorder (BIP), major depressive disorder (MDD), and schizophrenia (SCZ) [16, 17]. Bidirectional MR studies have also examined sleep apnea in relation to selected psychiatric disorders [18]. However, previous analyses have generally focused on individual sleep phenotypes or selected sleep-psychiatric disorder relationships. A systematic comparison of insomnia symptoms, sleep apnea, and sleep duration across a broad range of psychiatric disorders within a consistent univariable and multivariable MR framework remains limited.

Among various sleep-related phenotypes, insomnia symptoms, sleep apnea, and sleep duration have been extensively investigated using large-scale GWAS data [19], have phenotype-specific single-nucleotide polymorphism (SNP)-based heritability estimates of approximately 4% for self-reported insomnia symptoms, 22% for the MTAG-derived sleep apnea phenotype, and 9.8% for sleep duration [6, 20–22], and have established clinical relevance to psychiatric outcomes [23]. These three phenotypes capture distinct but complementary dimensions of sleep health: self-reported insomnia symptoms, sleep-disordered breathing, and sleep quantity.

Building on previous MR studies of individual sleep-psychiatric disorder relationships, we aimed to systematically compare the potential effects of three sleep-related phenotypes – self-reported insomnia symptoms, sleep apnea, and sleep duration – across 10 major psychiatric disorders using a consistent two-sample MR framework. We further applied MVMR to estimate the independent associations of these correlated sleep-related phenotypes when modeled simultaneously.

## Methods

### Study Design

A two-sample MR study was conducted to investigate the potential causal relationship between three sleep-related phenotypes and 10 psychiatric disorders. MR employs genetic variants as IVs to strengthen causal inference by minimizing confounding and reverse causation. The primary analyses evaluated the associations of genetically predicted insomnia symptoms, sleep apnea, and sleep duration with psychiatric disorders, in accordance with the STROBE-MR guideline. The completed STROBE-MR checklist is provided in the online supplementary material (for all online suppl. material, see https://doi.org/10.1159/phg/aenag005). The robustness of the findings was further assessed using MR-PRESSO and MVMR. A schematic of the overall study design is presented in Figure 1.

**Fig. 1.**
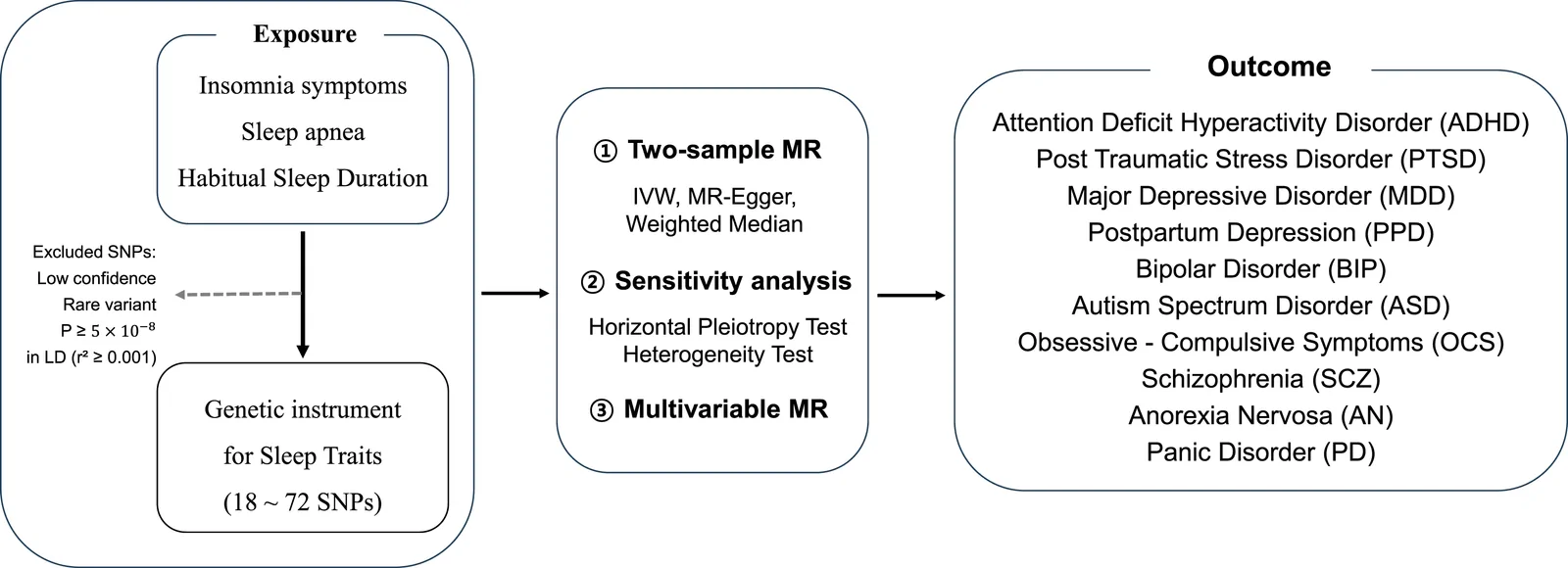
Flowchart of the study design and MR analysis.

### Data Source

Three sleep-related phenotypes were considered as exposures: self-reported insomnia symptoms, sleep apnea, and habitual sleep duration. Summary statistics for self-reported insomnia symptoms were obtained from a large-scale genome-wide association study (GWAS) of self-reported insomnia symptoms in more than 283,595 participants of European ancestry in the UK Biobank [20]. Insomnia symptoms (sleeplessness/insomnia; Data-Field 1200) were assessed using a self-report question asking whether participants had trouble falling asleep at night or woke up during the night, with response options “never/rarely,” “sometimes,” and “usually” (and “prefer not to answer”). Sleep-apnea summary statistics were derived from a meta-analysis of 159,255 individuals across five cohorts, including the UK Biobank [21]. Sleep apnea cases were ascertained using participant-reported diagnosis or EHR-based ICD codes (ICD-9: 327.23; ICD-10: G47.3), and in CLSA and AGDS the phenotype was defined based on responses to the item “stop breathing during sleep.” Summary statistics for habitual sleep duration were obtained from a recent UK Biobank GWAS that applied large-scale GWAS in 446,118 European participants [22]. In this GWAS, habitual sleep duration was assessed in UK Biobank at baseline by self-report as the integer number of hours slept per 24 h (including naps), with extreme responses (<3 or >18 h) excluded and “do not know/prefer not to answer” responses set to missing. Regarding effect size interpretation, self-reported insomnia symptoms and sleep apnea were analyzed as binary phenotypes. Therefore, all reported ORs represent the effect per unit increase in the log odds of genetic liability to each trait. Habitual sleep duration is a continuous trait measured in hours, and the reported ORs represent the effect per 1-h increase in genetically predicted sleep duration. For psychiatric outcomes, we selected the most recent and largest publicly available summary-level GWAS datasets from the Psychiatric Genomics Consortium (PGC) and affiliated consortia [24–33]. The ten outcomes included attention-deficit/hyperactivity disorder (ADHD), post-traumatic stress disorder (PTSD), MDD, postpartum depression (PPD), BIP, ASD, obsessive-compulsive symptoms (OCS), SCZ, anorexia nervosa (AN), and panic disorder. Detailed characteristics of the GWAS datasets used in this study are summarized in Table 1.

**Table 1.** Summary of GWAS datasets for sleep-related phenotypes and psychiatric disorders included in the MR analysis

Trait	Phenotype	GWAS consortium	Sample size (cases/controls)	Male/female numbers	Ancestry	GWAS catalog ID Or data DOI	Original GWAS citation	PMID
Exposure	Insomnia	UK Biobank	283,595(N/A)	NR	European	GCST90267286	Schoeler et al. 2023 [20]	37,106,081
​	Sleep apnea	MTAG (meta-analysis*)	159,255(N/A)	NR	European	GCST90267362	Campos et al. 2023 [21]	36,525,587
​	Sleep duration	UK Biobank	446,118(N/A)	NR	European	GCST007561	Dashti et al. 2019 [22]	30,846,698
Outcome	ADHD	PGC + iPSYCH + deCODE	225,534 (38,691/186,843)	NR	European	10.6084/m9.figshare.22564390	Demontis et al. 2023 [24]	36,702,997
​	PTSD	PGC	1,222,882 (137,136/1,085,746)	Sex-specific effects analyzed in paper but exact M/F N not extracted	European	10.6084/m9.figshare.26349322	Nievergelt et al. 2024 [25]	38,637,617
​	MDD	PGC	3,887,532 (525,000/3,362,532)	NR	European	10.6084/m9.figshare.27061255	Adams et al. 2025 [26]	39,814,019
​	PPD	PGC	70,765 (17,339/53,426)	Female only	European	10.6084/m9.figshare.24204843	Guintivano et al. 2023 [27]	37,849,304
​	BIP	PGC	840,309 (59,287/781,022)	NR	European	10.6084/m9.figshare.27216117	O'Connell et al. 2025 [28]	39,843,750
​	ASD	PSYCH + PGC	46,350 (18,381/27,969)	NR	European	10.6084/m9.figshare.14671989	Grove et al. 2019 [29]	30,804,558
​	OCS	PGC	33,943(N/A)	NR	European	10.6084/m9.figshare.26352157	Strom et al. 2024 [30]	38,548,983
​	SCZ	PGC	320,404 (76,755/243,649)	NR	European	10.6084/m9.figshare.19426775	Trubetskoy et al. 2022 [31]	35,396,580
​	AN	PGC	72,517 (16,992/55,525)	NR	European	10.6084/m9.figshare.14671980	Watson et al. 2019 [32]	31,308,545
​	PD	PGC	9,907 (2147/7760)	NR	European	10.6084/m9.figshare.16602218	Forstner et al. 2021 [33]	31,712,720

All GWAS summary statistics used in this study were downloaded in June 2025.

*Meta-analysis cohorts: UK Biobank; FinnGen; CLSA; AGDS; Partners Biobank.

### Selection of Instrumental Variable

SNPs associated with each exposure at genome-wide significance (*p* < 5 × 10^−8^) were selected as candidate instruments. To ensure independence among variants, linkage disequilibrium clumping was performed using PLINK v1.9 with an r^2^ threshold of < 0.001 and a 10 Mb window, based on the 1000 Genomes European reference panel. Instrument strength was evaluated using F-statistics, and weak instruments (F <10) were excluded. Prior to MR analyses, exposure and outcome summary statistics were harmonized to align effect alleles, and palindromic SNPs with intermediate allele frequencies (minor allele frequency > 0.42) were removed. The final number of SNPs included in each analysis varied depending on SNP availability across outcome datasets, and full lists of instruments are provided in online supplementary Table S1. The mean F statistics for selected instruments exceeded 39 across all analyses, indicating that weak instrument bias was unlikely. Although partial sample overlap cannot be fully excluded, the use of strong instruments (F >10) reduces bias toward observational associations.

### Statistical Analysis: Univariable MR and Sensitivity Analyses

Two-sample MR analyses were performed to evaluate the potential associations between sleep-related phenotypes and psychiatric disorders. The F statistics for univariable analyses were 39.67 for insomnia, 39.93 for sleep apnea, and 39.81 for sleep duration, all substantially exceeding the weak instrument threshold (F >10). The primary analytic approach was the inverse-variance weighted (IVW) method under a random-effects model, which provides a consistent estimate when all genetic variants are valid instruments. Robustness of the results was further assessed using complementary MR methods, including weighted median, MR-Egger regression, and mode-based estimators (simple and weighted mode). Horizontal pleiotropy was tested using the MR-Egger intercept, and potential outlier variants were identified and corrected through the MR-PRESSO global test. Heterogeneity across variants was quantified by Cochran’s Q statistic and the I^2^ index. Sensitivity analyses included leave-one-out tests, in which each variant was iteratively excluded to assess its influence on overall estimates. Visualization of results was provided through forest and scatter plots. To further assess causal directionality, the MR Steiger test was implemented. Reverse-direction MR analyses were additionally conducted using psychiatric disorders as exposures and the three sleep-related phenotypes as outcomes. IVW or the Wald ratio was used according to the number of instruments, with the same instrument-selection criteria and Bonferroni threshold as in the forward analyses.

### MVMR and Multiple-Testing Correction

MVMR was performed to assess the causal effects. Random-effects multivariable inverse-variance weighted analysis was used as the primary MVMR method. MVMR-Egger regression was additionally performed as an alternative method, and the MVMR-Egger intercept was examined to assess directional horizontal pleiotropy. To assess instrument strength in the MVMR framework, we calculated conditional F statistics for each exposure, which account for the correlation among sleep traits. In MVMR analyses, the conditional F statistics were below 10, indicating potential conditional weak-instrument bias; therefore, the MVMR estimates were interpreted cautiously as exploratory findings. Bonferroni correction was applied to evaluate statistical significance under multiple testing in the primary analyses. All analyses were conducted using the TwoSampleMR and MR-PRESSO packages in R (version 4.3.2).

### Ethical Considerations

This study used only publicly available summary-level GWAS data, with no access to individual-level information. The Institutional Review Board of Sungkyunkwan University determined that the study was exempt from ethical approval (IRB No. 2026-02–004). All original GWAS had obtained informed consent and ethical approval.

## Results

### Causal Effects of Sleep-Related Phenotypes on Psychiatric Disorders

Genetically predicted sleep-related phenotypes showed associations with the risk of developing several psychiatric disorders (Fig. 2). To account for multiple testing, we applied Bonferroni correction with a significance threshold of *p* < 0.0017 (0.05/30 tests: 3 exposures × 10 outcomes) (online suppl. Table S2).

**Fig. 2.**
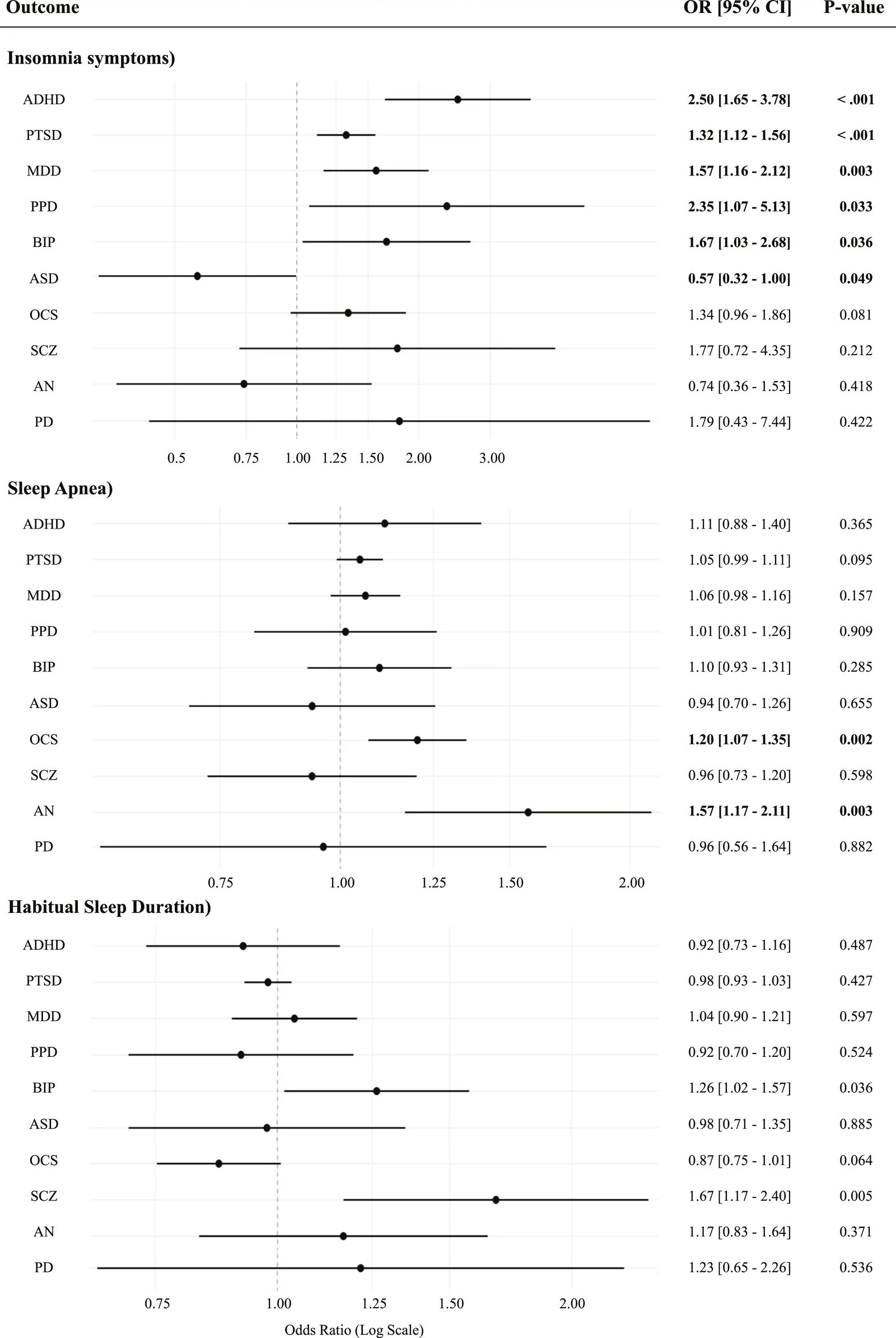
Univariable IVW MR forest plot for the associations between sleep-related phenotypes and psychiatric disorders.

Genetically predicted insomnia showed Bonferroni-significant associations with an increased risk of ADHD (OR 2.50, 95% CI 1.65–3.78, *p* < 0.001) and PTSD (OR 1.32, 95% CI 1.12–1.56, *p* < 0.001). Nominal associations were observed for MDD (OR 1.57, 95% CI 1.16–2.12, *p* = 0.003), PPD (OR 2.35, 95% CI 1.07–5.13, *p* = 0.033), and BIP (OR 1.67, 95% CI 1.03–2.68, *p* = 0.036). Conversely, an inverse association was observed for ASD, where genetically predicted insomnia symptoms showed an inverse association (OR 0.57, 95% CI 0.32–1.00, *p* = 0.049).

Genetically predicted sleep apnea showed nominal associations with increased risk of OCS (OR 1.20, 95% CI 1.07–1.35, *p* = 0.002) and AN (OR 1.57, 95% CI 1.17–2.11, *p* = 0.003), while no statistically significant associations were observed with other psychiatric disorders. Genetically predicted longer sleep duration showed nominal associations with BIP (OR 1.26, 95% CI 1.02–1.57, *p* = 0.036) and SCZ (OR 1.67, 95% CI 1.17–2.40, *p* = 0.005).

### Pleiotropy and Instrument Consistency in MR Analyses

Comprehensive sensitivity analyses were conducted to examine the influence of outliers of horizontal pleiotropy and heterogeneity across mental illness outcomes (Table 2). The MR-Egger intercept tests did not detect statistically significant evidence of directional horizontal pleiotropy in the univariable MR analyses (all Egger intercept *p* values >0.05). Cochran’s Q-statistic analysis revealed significant heterogeneity in several outcomes, including PTSD, MDD, and SCZ, associated with insomnia (*p* < 0.001).

**Table 2.** Univariable MR sensitivity analysis of sleep-related phenotypes

Exposure	Outcome	Cochran’s Q	*q* value	I^2^ (%)	MR-Egger intercept	Egger *p* value	PRESSO global *p*
Insomnia	ADHD	22.92	0.152	25.83	−0.007	0.480	0.169
​	PTSD	103.68	<0.001	83.60	0.006	0.075	<0.001
​	MDD	129.12	<0.001	86.83	0.007	0.270	<0.001
​	PPD	36.62	0.004	53.58	0.00	0.875	0.001
​	BIP	46.99	<0.001	63.82	0.005	0.677	0.001
​	ASD	18.77	0.342	9.43	−0.008	0.538	0.3534
​	OCS	19.57	0.297	13.14	0.00	0.697	0.347
​	SCZ	124.85	<0.001	86.38	0.018	0.372	<0.001
​	AN	32.25	0.014	47.29	−0.018	0.284	0.014
​	PD	17.92	0.394	5.16	0.003	0.919	0.389
Sleep apnea	ADHD	97.77	<0.001	65.23	−0.018	0.197	<0.001
​	PTSD	147.64	<0.001	77.65	0.004	0.267	<0.001
​	MDD	139.40	<0.001	75.61	0.004	0.391	<0.001
​	PPD	46.038	0.100	23.98	−0.006	0.646	0.125
​	BIP	88.41	<0.001	60.41	0.013	0.20	<0.001
​	ASD	72.2	<0.001	52.91	−0.033	0.059	<0.001
​	OCS	31.26	0.554	0.00	−0.001	0.848	0.584
​	SCZ	139.60	<0.001	74.93	−0.002	0.88	<0.001
​	AN	73.99	<0.001	54.05	0.019	0.304	<0.001
​	PD	28.99	0.712	0.00	0.012	0.709	0.735
Sleep duration	ADHD	135.10	<0.001	51.89	−0.003	0.633	<0.001
​	PTSD	224.73	<0.001	71.08	−0.001	0.578	<0.001
​	MDD	540.44	<0.001	88.71	−0.001	0.910	<0.001
​	PPD	103.63	0.007	31.48	−0.004	0.615	0.005
​	BIP	228.54	<0.001	68.93	0.009	0.221	<0.001
​	ASD	139.58	<0.001	50.57	−0.013	0.217	<0.001
​	OCS	74.85	0.167	14.49	−0.006	0.171	0.175
​	SCZ	430.42	<0.001	84.20	0.010	0.387	<0.001
​	AN	143.54	<0.001	54.02	−0.005	0.616	<0.001
​	PD	67.42	0.428	2.11	−0.004	0.851	0.437

The MR-PRESSO global test identified potential outliers in multiple analyses (*p* < 0.05). Outliers were detected for insomnia in most outcomes except ADHD, ASD, OCS, and panic disorder, and sleep apnea was also significant in most outcomes. Sleep duration also showed significance in analyses with some psychiatric outcomes, suggesting the possible influence of outliers. Causal effects were therefore re-estimated after removal of MR-PRESSO-identified outlier variants (outlier-corrected).

The outlier-corrected MR-PRESSO analysis demonstrated that insomnia significantly increased the risk of PTSD (OR 1.31, 95% CI 1.13–1.51, *p* = 0.004) and MDD (OR 1.40, 95% CI 1.13–1.72, *p* = 0.010). Similarly, the effects of sleep apnea on OCS (OR 1.20, 95% CI 1.07–1.35, *p* = 0.003) and AN (OR 1.57, 95% CI 1.17–2.11, *p* = 0.005), as well as the effects of sleep duration on BIP (OR 1.26, 95% CI 1.02–1.57, *p* = 0.039) and SCZ (OR 1.67, 95% CI 1.17–2.40, *p* = 0.007), maintained statistical significance after outlier correction.

Scatterplots of SNP-outcome associations and regression lines for each MR method (IVW, MR-Egger, weighted median, simple/weighted mode) are presented in online supplementary Figure S1, generally showing consistency in directionality with the IVW estimates (online suppl. Table S3). MR Steiger analyses generally supported the hypothesized exposure-to-outcome direction, and the leave-one-out analyses indicated that most estimates were not substantially driven by any single genetic variant. Detailed results are presented in online supplementary Table S4. Reverse-direction MR was estimable for 19 of the 30 planned associations. After Bonferroni correction, MDD and PTSD were associated with insomnia symptoms and sleep apnea, whereas no significant reverse association was observed for sleep duration or with BIP or SCZ as exposures (online suppl. Table S6).

### MVMR of Sleep-Related Phenotypes on Psychiatric Disorders

MVMR was performed to estimate the independent associations of correlated sleep-related phenotypes. A comparison of the univariable IVW and MVMR results is presented in Figure 3. MVMR was used to evaluate the direct associations of insomnia symptoms, sleep apnea, and sleep duration on psychiatric disorders after adjustment for the other sleep-related phenotypes. The same Bonferroni-corrected significance threshold (*p* < 0.0017) was applied.

**Fig. 3.**
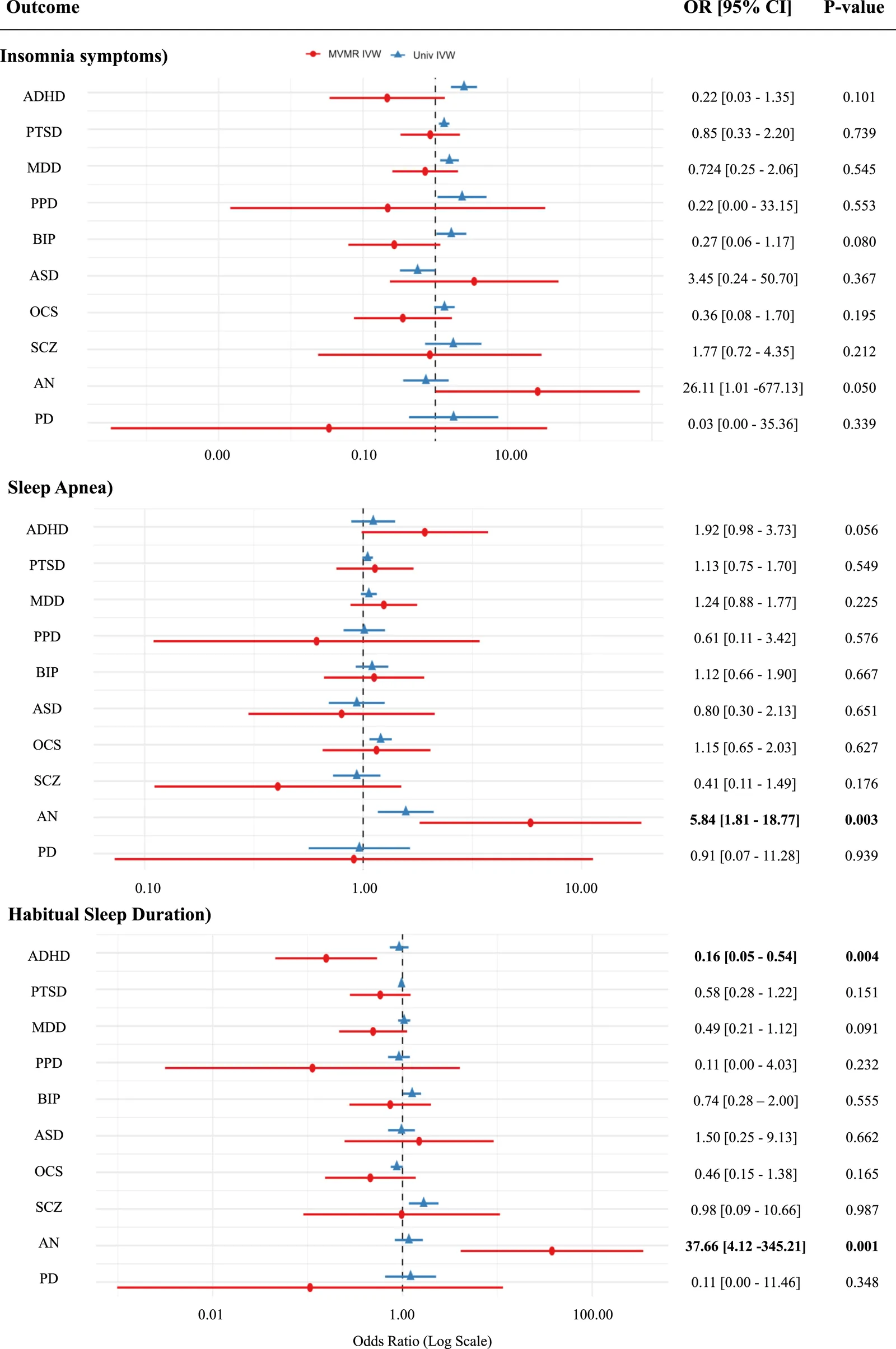
Forest plot comparing univariable IVW and multivariable IVW (MVMR) estimates for sleep-related phenotypes and psychiatric disorders.

In the MVMR analysis, the association between sleep apnea and AN was maintained (OR 5.84, 95% CI 1.81–18.77, *p* = 0.003) whereas no significant associations were observed for other outcomes. Sleep duration showed a nominal inverse association with ADHD (OR 0.16, 95% CI 0.05–0.54, *p* = 0.004) and a positive association with AN that met the Bonferroni-corrected significance threshold (OR 37.66, 95% CI 4.12–345.21, *p* = 0.001). However, the extremely wide confidence interval for the sleep duration-AN association suggests substantial uncertainty in the effect estimate, likely attributable to limited overlap in IVs across correlated sleep-related phenotypes and reduced conditional instrument strength in the MVMR framework.

Conversely, for insomnia, significant associations were not confirmed for most outcomes. Additional MVMR-Egger sensitivity analyses are presented in online supplementary Figure S2. The MVMR-Egger estimates were generally imprecise, with wide confidence intervals, and the conditional F statistics were below 10 for all three sleep-related phenotypes, indicating potential conditional weak-instrument bias. Detailed MVMR-Egger estimates, Egger intercepts, conditional F statistics, and modified Q statistics are provided in online supplementary Table S5. Therefore, the MVMR findings should be interpreted cautiously as exploratory results.

## Discussion

This study extends previous MR evidence by systematically comparing the associations of three sleep-related phenotypes – self-reported insomnia symptoms, sleep apnea, and sleep duration – with 10 psychiatric disorders within a consistent analytical framework. In addition, MVMR was applied to examine whether these correlated sleep-related phenotypes showed independent associations when modeled simultaneously. After applying Bonferroni correction for multiple testing, the most robust associations were observed between insomnia and ADHD/PTSD, with an additional suggestive association between sleep duration and AN observed in the exploratory MVMR analysis. The key findings were as follows. Bonferroni-significant associations were observed between insomnia and ADHD and PTSD, whereas nominal associations were identified for MDD, PPD, and BIP. Genetically predicted sleep apnea showed nominal associations with OCS and AN, and longer sleep duration showed nominal associations with BIP and SCZ. Overall, these findings extend the existing MR literature by providing a systematic assessment of multiple sleep-related phenotypes across a broad range of psychiatric disorders, although the strength of evidence varied across the evaluated associations.

Among the evaluated sleep-related phenotypes, genetically predicted insomnia symptoms showed associations with the broadest range of psychiatric outcomes. The significant associations between insomnia symptoms and ADHD, PTSD, MDD, PPD, and BIP are consistent with previous observational studies [34, 35], and this study provides genetic evidence that is less susceptible to reverse causation. Previous MR studies have reported associations of insomnia symptoms with selected psychiatric outcomes, including ASD, BIP, MDD, and SCZ [36, 37]. Consistent with and extending this evidence, our study evaluated insomnia alongside sleep apnea and sleep duration across 10 psychiatric disorders within the same analytical framework, enabling comparison of the magnitude and consistency of associations across outcomes.

Several biological pathways have been proposed as potential mechanisms linking insomnia to mental illness. First, chronic insomnia induces hyperactivation of the hypothalamic-pituitary-adrenal axis, increasing 24-h cortisol secretion [38], which is associated with impaired emotional regulation and stress response. Second, insomnia causes dysregulation of the monoamine neurotransmitter system (serotonin, dopamine, and norepinephrine) [39], which is linked to the core pathophysiology of mood disorders and ADHD. Third, sleep deprivation increases neuroinflammation [40], which leads to structural and functional changes in the hippocampus and prefrontal cortex, leading to emotional dysregulation and cognitive decline [41]. Fourth, insomnia has been suggested to impair emotional and cognitive regulation and disrupt circadian rhythms [10]. Meanwhile, the inverse signal observed in ASD requires caution in interpretation due to phenotypic heterogeneity, differences in insomnia definition and measurement, or the possibility of pleiotropy of some SNPs, and further validation studies are needed. The reverse-direction findings also suggest potential bidirectionality, particularly between PTSD and insomnia symptoms, warranting cautious interpretation of the forward associations.

Sleep apnea showed nominal associations with OCS and AN among 10 psychiatric disorders. While previous MR studies have primarily focused on the relationship between sleep apnea and MDD and SCZ [42], this study provides additional evidence in that it highlights the outcome specific associations with OCS and AN. Several mechanisms have been proposed by which sleep apnea influences OCS and AN. First, intermittent hypoxia induces dysfunction of the fronto-striatal circuitry [43, 44], which is associated with repetitive thoughts and compulsive behaviors, which are core pathologies of OCS. Second, oxidative stress and neuroinflammation caused by hypoxia may impair cognitive control ability [45], which may worsen OCS. Third, the association between sleep apnea and AN may be explained by a pathway in which endocrine changes, such as disruption of the leptin-ghrelin axis, affect eating behavior mediated by common risk factors such as obesity and metabolic abnormalities [46]. The association between sleep apnea and AN remained nominally significant in the MVMR analysis; however, this finding should be interpreted cautiously given the limited precision and potential conditional weak-instrument bias.

Genetically predicted longer sleep duration was associated with an increased risk of BIP and SCZ. This suggests a reinterpretation of the commonly held belief that a longer sleep duration leads to better health from a genetic perspective. While previous MR studies have reported an association between sleep duration and bipolar disorder [26, 47], this study further explored the relationship with SCZ. The mechanisms by which long sleep duration is associated with an increased risk of BIP and SCZ may be interpreted as follows. First, hypersomnia may be a prodromal symptom of BIP and SCZ, reflecting sleep-wake cycle dysregulation that predates the onset of the disease. Furthermore, long sleep duration is associated with circadian rhythm dysregulation [48], a key pathophysiology of BIP and SCZ. Additionally, a shared genetic vulnerability may exist. That is, long sleep duration and psychiatric disorders may share common genetic variants and thus are not independent of each other. In the MVMR analysis, sleep duration was inversely associated with ADHD and positively associated with AN, with only the AN association meeting the Bonferroni-corrected significance threshold. However, the extremely wide confidence interval and low conditional instrument strength indicate substantial uncertainty in this estimate. Therefore, the magnitude and independence of this association should not be interpreted as conclusive.

The attenuation of the insomnia-symptom associations in MVMR may partly reflect the substantial overlap and multidimensional nature of sleep-related phenotypes [48]. Notably, the sleep duration–AN association remained significant after Bonferroni correction in MVMR, although this finding should be interpreted cautiously because of its wide confidence interval and reduced conditional instrument strength. Indeed, sleep health has been reported to be influenced by multiple dimensions, including sleep duration, continuity, efficiency, timing, and regularity, and unhealthy sleep-related phenotypes can coexist [49, 50]. Furthermore, insomnia, sleep apnea, and sleep duration show significant clinical overlap [51], and evaluating only a single sleep trait may confound the effects of other sleep-related phenotypes. The MVMR results suggest that overall sleep dysregulation, rather than individual sleep-related phenotypes, is important for the risk of mental disorders. In other words, it is clinically more important to comprehensively assess and intervene on sleep quantity, quality, and breathing, rather than focusing solely on improving insomnia [52]. Furthermore, some weakening of associations may be due to the limited number of common IVs in the MVMR analysis or to reduced estimation precision due to multicollinearity issues [53, 54]. Conditional F statistics in MVMR were below 10, indicating potential conditional weak-instrument bias when jointly modeling the correlated exposures. Therefore, the MVMR findings should be regarded as exploratory and interpreted cautiously when assessing the independent associations of individual sleep-related phenotypes.

The results of this study suggest that different sleep-related phenotypes may be relevant to psychiatric risk and warrant further investigation as potential targets for risk assessment and intervention. These findings support the need for integrated sleep management, including early sleep assessment, screening for sleep apnea, and monitoring for extremes (over/under) of sleep duration in individuals at risk for mental disorders. First, early sleep assessment is essential in high-risk groups for mental disorders. Early detection and treatment of insomnia, particularly in individuals at high risk for ADHD, PTSD, and MDD, may be clinically relevant to mental disorders. Second, screening for sleep apnea is important in patients with OCS and AN. Assessing sleep apnea and providing treatments such as CPAP in these patients may contribute to improving psychiatric symptoms. Third, monitoring sleep patterns may be informative in patients with BIP and SCZ as changes in sleep duration including hypersomnia could reflect clinical deterioration or relapse risk. Finally, a precision psychiatric approach is possible. Genetic risk scores can be used to predict an individual’s vulnerability to sleep problems and mental illness, allowing for the development of personalized preventive strategies [55]. Furthermore, recent scientific recommendations that sleep health should be defined multidimensionally and managed within a standardized framework provide room for future integration of sleep indicators into risk stratification and prevention strategies in precision psychiatry.

A strength of this study is the use of large-scale GWAS data, which maximizes statistical power. Furthermore, a two-sample MR design was employed to minimize the influence of reverse causality and confounding variables. Various sensitivity analyses (MR-Egger, weighted median, MR-PRESSO) were performed to confirm the robustness of the results. A further strength of this study is the systematic comparison of three correlated sleep-related phenotypes across 10 psychiatric disorders within a consistent univariable and multivariable MR framework. This approach extends previous studies that primarily examined individual sleep phenotypes or selected sleep-psychiatric disorder relationships. However, this study has several limitations. First, the GWAS data used in this study primarily originate from individuals of European ancestry, limiting the generalizability of our findings to other ethnic groups. Second, insomnia symptoms and sleep duration were primarily self-reported, while sleep apnea was not uniformly confirmed by polysomnography across cohorts. Third, MR analysis assumes linear effects, making it impossible to assess nonlinear relationships. Fourth, the phenotypic heterogeneity of mental disorders makes it difficult to distinguish effects across disease subtypes. Fifth, several MVMR estimates, particularly the association between sleep duration and AN, had extremely wide confidence intervals. Moreover, the conditional F statistics were below 10, indicating that the estimates may have been affected by conditional weak-instrument bias. Therefore, the MVMR findings should be considered exploratory rather than conclusive evidence of independent causal effects. Future studies should complement the limitations of this study by further verifying whether genetic causality estimates lead to actual intervention effects through nonlinear MR, GWAS including racial and ancestral diversity, and linking with clinical intervention studies.

## Conclusion

This study genetically identified the potential causal link between sleep-related phenotypes and mental illness through MR analysis using large-scale GWAS data. Insomnia showed Bonferroni-significant associations with ADHD and PTSD, whereas the associations involving MDD, PPD, BIP, OCS, AN, and SCZ were nominal and require further validation. Overall, the findings indicate that different sleep-related phenotypes may show distinct patterns of association with psychiatric outcomes and highlight the value of evaluating multiple dimensions of sleep within a unified analytical framework.

## Acknowledgments

The authors thank the investigators and participants of the original GWAS studies for generating and sharing the summary-level data analyzed in this work. We also acknowledge the GWAS Catalog as well as the Psychiatric Genomics Consortium (PGC) and GWAS Catalog for providing open access to the relevant GWAS summary statistics.

## Statement of Ethics

This study used only publicly available summary-level GWAS data, with no access to individual-level information. The Institutional Review Board of Sungkyunkwan University determined that the study was exempt from ethical approval (IRB No. 2026-02-004). The requirement for informed consent was waived by the Institutional Review Board of Sungkyunkwan University. The study was conducted in accordance with the World Medical Association Declaration of Helsinki. All original GWAS had obtained informed consent and ethical approval.

## Conflict of Interest Statement

The authors declared no potential conflicts of interest with respect to the research, authorship, and/or publication of this article.

## Funding Sources

This research was supported by a grant of the Korea Health Technology R&D Project through the Korea Health Industry Development Institute (KHIDI), funded by the Ministry of Health & Welfare, Republic of Korea (RS-2025-02309552). This research was also supported by the Ministry of Education, Ministry of Science & ICT, and Ministry of Health and Welfare, Republic of Korea (grant numbers: NRF [2021-R1-I1A2 (059735)], RS [2024-0040 (5650)], RS [2019-II19 (0421)], RS [2025-2544 (3209)], RS [2020-II20 (1821)], RS [2026-2561 (0094)], RS [2026-2561 (5334)], and RS [2026-2561 (3012)].

## Author Contributions

Gahyun Kim and Minyoung Kim: data analysis; Gahyun Kim, Kyung-Hun Sung, Youngoh Bae, and Seung Won Lee: drafting and revising the article; Youngoh Bae and Seung Won Lee: final approval of the version to be published and agreed to be accountable for all aspects of the work.

## Data Availability

The summary-level data on insomnia, sleep apnea, and sleep duration were obtained from the GWAS catalog database (https://www.ebi.ac.uk/gwas/). The summary-level GWAS data for the ten psychiatric outcomes were obtained from the Psychiatric Genomics Consortium (PGC) (https://pgc.unc.edu/).
